# Fiber-Reinforced Cemented Paste Backfill: The Effect of Fiber on Strength Properties and Estimation of Strength Using Nonlinear Models

**DOI:** 10.3390/ma13030718

**Published:** 2020-02-05

**Authors:** Xin Chen, Xiuzhi Shi, Shu Zhang, Hui Chen, Jian Zhou, Zhi Yu, Peisheng Huang

**Affiliations:** 1 School of Resources and Safety Engineering, Central South University, Changsha, Hunan 410083, China; chenxin_ck@csu.edu.cn (X.C.); baopo@csu.edu.cn (X.S.); zhangshu@csu.edu.cn (S.Z.); chenhui@xju.edu.cn (H.C.); csujzhou@hotmail.com (J.Z.); 2 Fankou Lead-Zinc Mine, Shaoguan, Guangdong 512325, China; huangpeisheng_fk@163.com

**Keywords:** fiber-reinforced CPB, compressive strength, fiber reinforcement index, nonlinear model, estimate

## Abstract

This experimental investigation was conducted to research the properties of polypropylene (PP) fiber-reinforced cemented paste backfill (CPB). The unconfined compressive strength (UCS) of the fiber-reinforced CPB showed a significant improvement with average UCS increase ratios of 141.07%, 57.62% and 63.17% at 3, 7 and 28 days, respectively. The macroscopic failure mode and SEM analysis indicated that fibers prevented the formation of large tensile and shear cracks during the pull-out and pull-off failure modes. A linear fitting function for the UCS at a curing time of 3 days and two polynomial fitting functions for the UCS at curing times of 7 and 28 days were established to characterize the relationship between the UCS of the fiber-reinforced and unreinforced CPB. Moreover, based on composite mechanics, nonlinear models related to the UCS and fiber reinforcement index were obtained. The estimated functions containing the fiber reinforcement index *λ*, which consists of the fiber content and aspect ratio of fiber, could evaluate the UCS. Furthermore, the fiber reinforcement index *λ* quantifies the enhancement by the fibers. Both estimation results indicated that the UCS values were estimated accurately at curing times of 3, 7 and 28 days in this study. Additionally, the estimation models could be used to guide the strength design of fiber-reinforced CPB. Besides this, the results showed that fiber-reinforced CPB can be used more widely in mine backfills and meets the requirements of controlled low-strength material (CLSM) for broader applications.

## 1. Introduction

The treatment of mine tailings has become a hot topic in mining engineering research in recent years due to the potential environmental hazards as well as recovery value prospects [1,2]. Cemented paste backfill (CPB), a tailing disposal method, innovatively transfers tailings from surface storage to underground and has been widely accepted by numerous mining enterprises globally [3,4,5,6,7]. Not only is the environmental pollution by tailings reduced, but the method also provides support for underground voids. In general, CPB is a cement-based material with a certain strength formed by mixing cement, tailings and water, and it is commonly evaluated by its unconfined compressive strength (UCS), which ranges from 0.2–4 MPa [8]. As the binding agent, cement provides cohesive strength for the CPB, and the UCS increases with increasing cement content [8,9,10]. However, too much cement also incurs a high backfill cost, especially for tailings produced during mineral processing, which contain much finer aggregates and sulfides, causing a low strength of the CPB. A significant amount of cement is used to meet the strength requirements [11,12,13]. This requirement leads to the cement cost representing 50–75% of the cost of CPB preparation [14]. Therefore, reducing the cement consumption according to different backfill function requirements is an effective method to decrease mine backfilling costs. Besides, certain cement substitutes, such as fly ash and phosphogypsum, have been studied as binders for CPB preparation [15,16,17,18]. However, subject to the production and their inherent harmful factors, these materials can only reduce the cement consumption to a limited extent.

Fiber-reinforced cement-based materials are becoming more popular in civil and structural engineering because they are used to enhance the strengths of soils and structures by the high strength and ductility characteristics of the fiber material [19,20,21,22,23,24,25]. Tang et al. [26] reported that polypropylene (PP) could result in an increase in the UCS of soil with or without cement. Cristelo et al. [27] also indicated that PP fibers could improve the UCS of soil structures at all experimental cement content levels, and the fibers were also helpful in improving the stiffness and ductility of the material. Toutanji et al. [28] used 0.1–0.5 vol.% of fiber to prepare concrete, and the results showed that the fibers increased the permeability of conventional concretes. In addition, fiber-reinforced technology was also applied in mining engineering, mainly in the field of mine backfilling. In 1987, to reduce the cement usage, Mitchell and Stone [29] first used metal shotcrete fibers and anchored fibers to prepare backfill. The experimental results illustrated that the fibers could improve the strength of the CPB. Since then, scholars have also conducted related research to study the mechanism of fiber reinforcement in detail and expand the range of applications of fiber in mine backfills; it is generally accepted that fiber can enhance the UCS of backfill materials [10,30,31,32]. However, related research is still rare, specifically regarding the utilization of sulfur-containing fine tailings, which are difficult to dispose of.

In addition, due to differences in the properties of the engineering materials, such as the type of fiber, cement and tailing material, the effects of fiber reinforcement are different in their respective engineering applications, and their increases in structural strength range from dozens to hundreds. [30,32]. In previous studies, with the exception of comparing the strength changes by intuitive means, researchers have mainly demonstrated the enhancement via fiber addition by analyzing the macroscopic and SEM patterns, but it is difficult to quantitatively present the fiber reinforcement effect [27,32]. Therefore, a mathematical function is proposed and considered to be able to quantitatively describe the relationship between strength and fiber content. Fiber-reinforced cement-based materials are considered composite materials that meet the superposition principle of composite mechanics [33]. In fiber-reinforced concrete applications, a nonlinear formula related to the influence of fiber parameters was proposed as Equations (1) and (2), and a fiber reinforcement index of λ, consisting of the fiber volume fraction and aspect ratio, was used to quantify the effect of adding fiber [34,35]. In subsequent studies, the evaluation model of the UCS of concrete under the influence of a steel–polypropylene hybrid and steel fiber was developed, and good estimation results were obtained. In the field of mine backfilling, there are few related studies due to the differences in material properties and proportions of the soil and concrete used in practice.

(1) $$f_{fc}=f_{c}\;\left(1+\overset{n}{\underset{i=1}{\sum }}\alpha_{i}\lambda_{if}\right)$$

(2) $$\lambda_{if}=\rho_{if}\;\frac{l_{if}}{d_{if}}$$

where *f_fc_* and *f_c_* are the UCS of the fiber-reinforced concrete and unreinforced concrete, respectively; *λ_if_* is the fiber reinforcement index of the *i*th fiber; *ρ_i__f_* is the volume fraction of the *i*th fiber, vol.%; *l_if_*/*d_if_* is the aspect ratio of the *i*th fiber; *l_if_* is the length of the *i*th fiber; *d_if_* is the diameter of the *i*th fiber, mm; *α* is the influence factor of the fiber; *i* is the number of types of added fiber.

Based on the above considerations, experimental and nonlinear model studies were conducted to investigate the UCS of CPB. Firstly, the effect of fibers on the UCS of CPB was investigated through experimental data and macroscopic failure mode analysis. Then, the UCS of the fiber-reinforced CPB as a function of the UCS of the unreinforced CPB was studied with different fitting methods. Moreover, the effects on the CPB and failure modes of fibers were described with data comparison and microscopic analysis, and nonlinear modes were established based on composite mechanics to estimate the UCS of fiber-reinforced CPB and quantify the reinforcement by the fibers. Finally, the application prospects of fiber-reinforced CPB were discussed.

## 2. Materials and Methods 

### 2.1. Materials Characterization

**Figure 1:**
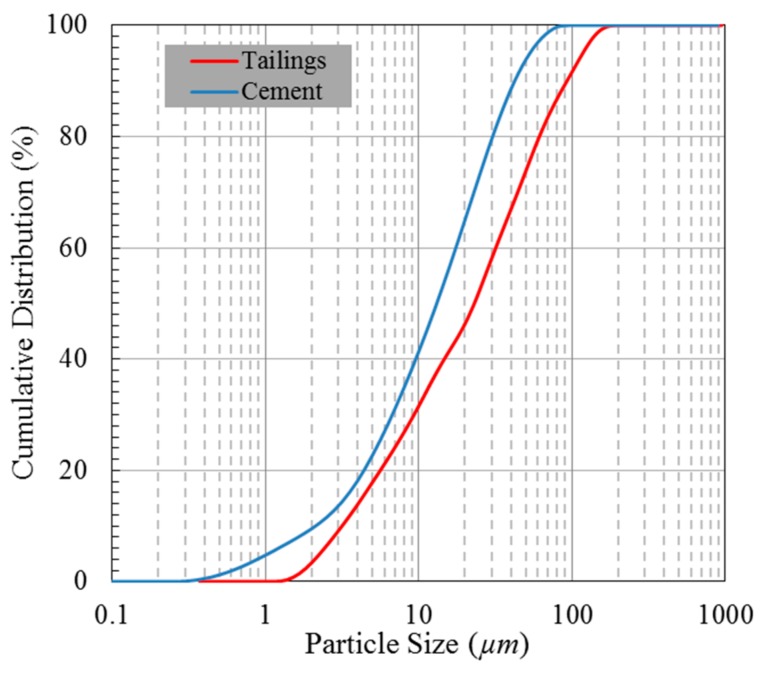
Particle size distribution of tailings and cement.

Tailings were collected in the Fan Kou lead-zinc mine in China. The particle size distributions of the tailings and cement were determined by a LS particle size analyzer (LS13320, Beckman, Brea, CA, USA). Particle diameter is determined by analyzing the light scattering and diffraction characteristics caused by particles. The size distribution of the tailings could be characterized by a median diameter of *D*_50_ = 25.31 μm, and more than 47.15% of the particles had a size that was smaller than 20 µm, which indicated that the tailings belonged to the medium particles [36]. Portland cement P.O 42.5R was used as the binder, which contains a large number of fine particles with a particle size smaller than 20 μm; their particle size distribution is shown in Figure 1. In addition, the physical properties and chemical compositions of tailings were tested by soil testing method specified by China’s standard [37] and X-ray fluorescence spectrum (XRF) (ZSX Primus II, Rigaku Corporation, Akishima-Shi Tokyo, Japan), respectively. XRF is a method that uses primary X-ray photons to excite the atoms in the substance to be measured and make them produce secondary X-rays to analyze the composition and study the chemical state of the substance. The results shown in Table 1 indicated that tailings contained much oxides and 11.90% sulfur ion compounds. Monofilament polypropylene (PP) fibers were used as an additive with a tensile strength and elastic modulus that were greater than 350 MPa and 3.5 GPa, respectively. The lengths of the fibers were approximately 3, 6, 9 and 12 mm.

### 2.2. Specimen Preparation and Test Method

**Figure 2:**
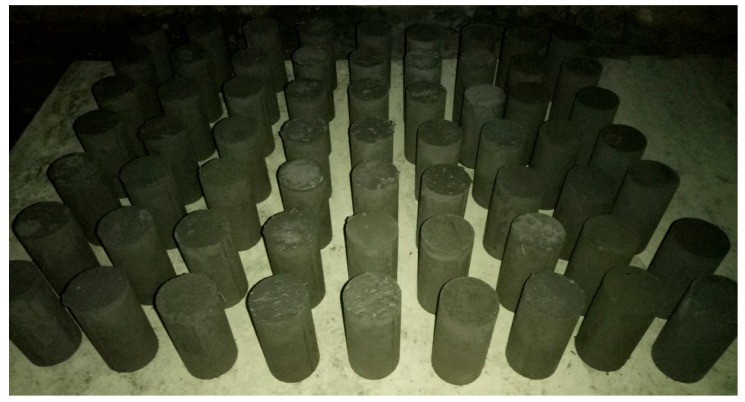
The cemented paste backfill (CPB) specimens for the unconfined compressive strength (UCS) test.

Orthogonal tests are an effective method of multi-factor analysis with a minimum of the computational load designed based on an orthogonal table. This method conducts tests by selecting a suitable number of representative test cases from many test data and has the advantages of even dispersal and neat comparability with the minimized trials for a complete analysis [38,39]. To study the effects of adding materials on the properties of CPB, as shown in Table 2, an orthogonal test of *L*_16_ (4^4^) based on orthogonal table was designed to investigate the effects of fibers and cement on the strength properties of CPB. The four factors used were the cement content (A), solid mass concentration (B), fiber content (C) and fiber length (D), with four levels for each factor as follows: 13 wt.% (A_1_), 10 wt.% (A_2_), 8 wt.% (A_3_) and 7 wt.% (A_4_); 60 wt.% (B_1_), 62 wt.% (B_2_), 64 wt.% (B_3_) and 66 wt.% (B_4_); 0.05 vol.% (C_1_), 0.11 vol.% (C_2_), 0.16 vol.% (C_3_) and 0.22 vol.% (C_4_); and 3 mm (D_1_), 6 mm (D_2_), 9 mm (D_3_) and 12 mm (D_4_), respectively. The size of the specimens was 50 mm in terms of diameter and 100 mm in terms of height, and triple specimens were prepared for each mixture at different curing ages, as shown in Figure 2. The raw materials were mixed for 5–10 min and poured into the molds, and the specimens were cured with temperature of 22 ± 1 °C and relative humidity of more than 90%. When the end of each curing time was reached, the UCSs of all mixtures were tested using a computer-controlled fully automatic pressure testing machine (WHY - 200, Shanghai Hualong Test Instruments Corporation, Shanghai, China). Specimens were loaded at a constant displacement rate of 0.2 mm/min, and the stress and displacement performance were recorded until each specimen was destroyed. New specimens were also prepared for the scanning electron microscope (SEM) (QUANTA FEG 250, FEI, Hillsborough, OR, USA) tests in the same manner as was used for the experimental mixtures to test the hydration products and microstructure. This method can directly use the material properties of sample surface materials for microscopic imaging. Before the test, specimens were made into millimeter-sized micro samples and gold plated.

## 3. Results and Discussion

### 3.1. The Effect of the PP Fiber on the UCS of CPB with Cement Less Than 13 wt.%

**Figure 3:**
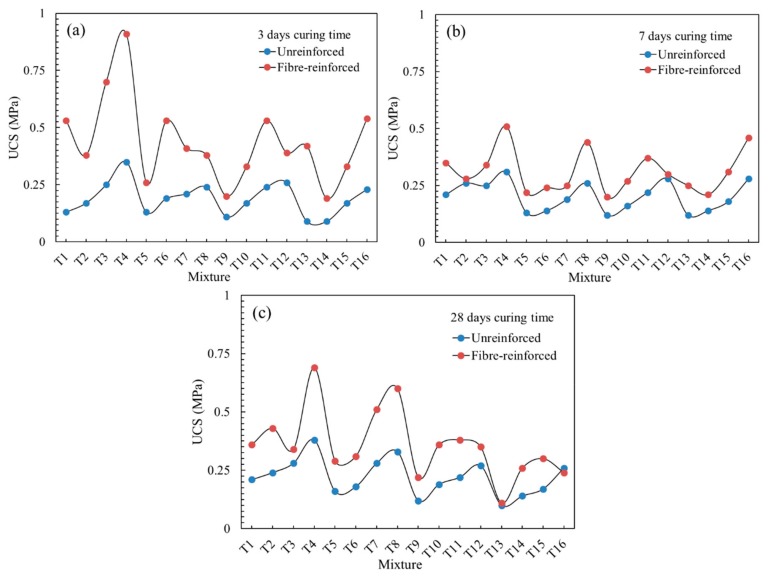
UCSs of the fiber-reinforced and unreinforced specimens with different curing times: (**a**) 3 days curing time; (**b**) 7 days curing time; (**c**) 28 days curing time.

As shown in Figure 3, the maximum UCS values of unreinforced CPB and fiber-reinforced CPB are 0.38 and 0.91 MPa, respectively. The results show that the main reason for the low compressive strength of the CPB is that the tailing’s particles are too fine [12,40]. The UCS development trend presented in Figure 3 demonstrated that with increasing cement content, the solid mass concentration and curing age were effective measures to improve the strength of the CPB. The highest UCS of unreinforced CPB was 0.38 MPa for the T4 mixture with a cement content of 13 wt.% and a solid mass concentration of 66 wt.% at a curing time of 28 days. A small part of CPB showed a slow increase or even a decrease in UCS with the extension of curing age. This result is because, in the case a high content of sulfur-containing tailings, the mixture is likely to adversely affect the strength of the CPB. Some hydrated gelling product was used to resist the sulfate attack, which weakened its effect on the strength of CPB [13]. Furthermore, the hydration secondary expansion products increasingly accumulate in the later stage of curing, causing the CPB to crack and reduce the strength.

For the fiber-reinforced CPB, the analysis of orthogonal test results is shown in Table 3, where *k_i_* represents the average of all UCS_fiber-reinforced_ under level *i*, and *R_j_* represents the range (the difference between the maximum and minimum average values) [41]. The significance indicates that the cement and solid mass concentration are still the most important factors affecting the UCS of CPB. Furthermore, the UCS of fiber-reinforced CPB increases with the increase of the cement and concentration. Even though all specimens had UCS values of less than 1 MPa, there still was a significant improvement, as shown in Figure 3. The following equation is used to calculate the increase ratio of the UCS of the fiber-reinforced CPB:

(3) $$R_{e}=\frac{\sigma_{fiber}-\sigma_{no-fiber}}{\sigma_{no-fiber}}\times 100\%$$

where *R_e_* is the UCS increase ratio, %; *σ_fiber_* is the UCS of the fiber-reinforced CPB, MPa; and *σ_no-fiber_* is the UCS of the unreinforced CPB, MPa.

**Figure 4:**
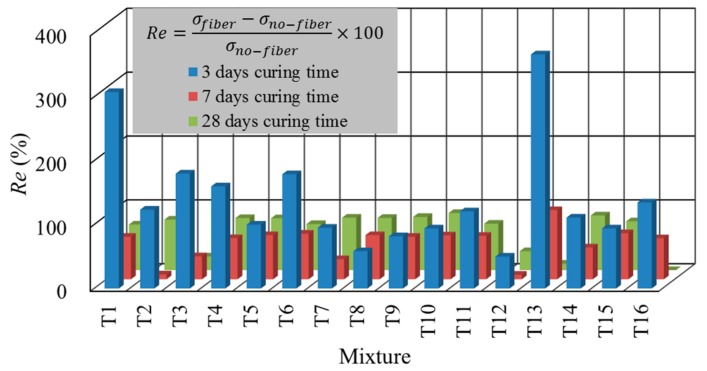
The UCS increases the ratio of the fiber-reinforced CPB.

Figure 4 indicates that the average UCS of the fiber-reinforced CPB with cement content of 7–13 wt.% increased by 141.07%, 57.62% and 63.17% at 3, 7 and 28 days, respectively. In the previous study, when cement content exceeded 13 wt.%, the average UCS of the fiber-reinforced CPB increased by 213.11%, 43.96% and 143.45% at 3, 7 and 28 days, respectively [32]. Due to the influence of the fiber, the strength was no longer completely developed according to the trend of high cement content, resulting in high strength. In the UCS tests of the fiber-reinforced CPB, the fibers provided additional strength after the failure of the backfill matrix. At a curing time of 3 days, fibers could fully exert their function of resisting pressure. However, the sulfur-containing tailings tended to cause a sulfate attack on the CPB by generating many secondary products such as ettringite and gypsum during the hydration reaction process, which have been reported to cause the decrease of UCS at the later stage of curing [13]. Under the action of expansion, as the curing time was prolonged, the CPB matrix would crack from the interior and expand outward, which may even have caused the matrix to disintegrate. To resist the development of cracks, the fibers were deformed and stretched to varying degrees before the test. Therefore, the fiber-reinforced CPB exhibited a poorer UCS at 7 and 28 days compared to at 3 days because the fiber could not fully exert its pressure resistance effect. Besides, when the cement content is higher than 14 wt.%, the UCS of CPB still increases significantly when curing 28 days, and small cement content (less than 13 wt.%) cannot completely eliminate the negative impact of a sulfate attack.

**Figure 5:**
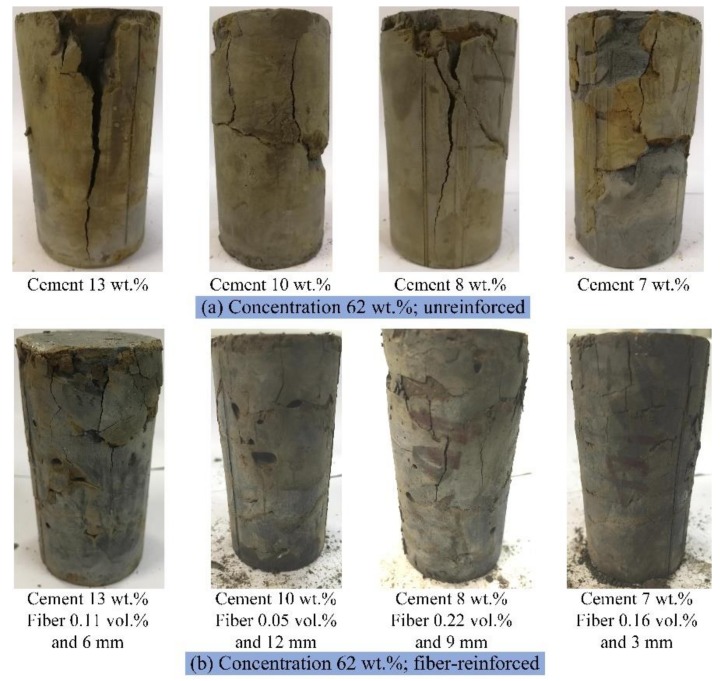
The macroscopic failure modes of the CPB specimens: (**a**) unreinforced with concentration of 62 wt.%; (**b**) fiber-reinforced with concentration of 62 wt.%.

The macroscopic failure modes of the eight CPB specimens are listed in Figure 5 to compare the different failure modes of the fiber-reinforced CPB and unreinforced CPB. As shown in Figure 5a, the main failure modes of the unreinforced CPB were tensile failure and shear failure, and the nearer to the loading area the failures were, the more serious the failures; similar results have also been reported in previous studies [42]. Several large cracks formed in the specimens under pressure; in Figure 5a, the specimens with 13 wt.% cement and 8 wt.% fiber even exhibited vertical transfixion cracks parallel to the loading direction, and the specimens with 10, 8 and 7 wt.% cement showed transverse shear cracks. These cracks eventually led to the disintegration of the specimens. In comparison, the fiber-reinforced CPB shown in Figure 5b only exhibited some small tensile cracks, and the specimens still maintained good integrity. This result indicates that the fibers could inhibit the development of cracks and even repair them, thereby reinforcing the strength of the CPB.

### 3.2. The Effect of Cement on the UCS

**Figure 6:**
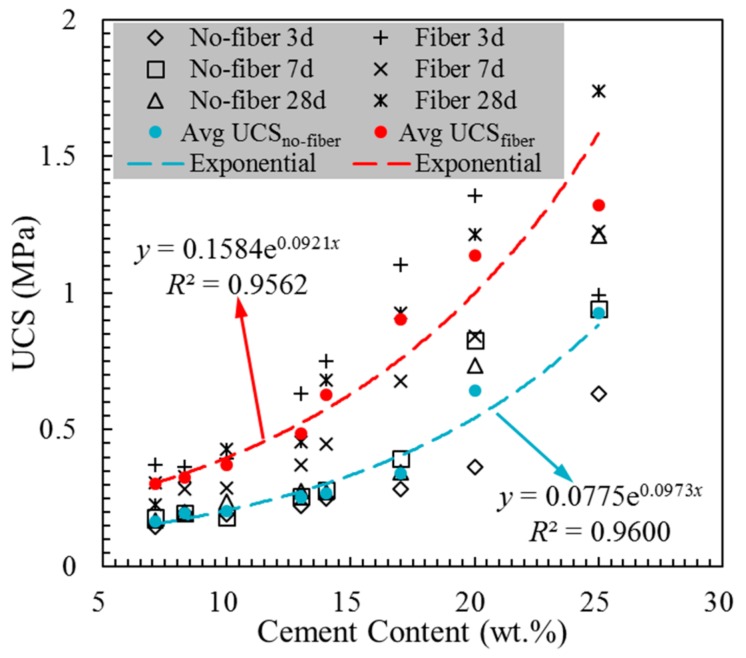
The effect of the cement content on the UCS.

The cement content played a significant role in enhancing the strength of the CPB. Therefore, in this study, the effects of eight different cement contents (25, 20, 17, 14, 13, 10, 8 and 7 wt.%) on the UCS of the fiber-reinforced and unreinforced CPB were studied. Some of the data have been published in previous studies [32]. Figure 6 shows the trend of the UCS versus cement content at three curing times. In general, as mentioned above, a higher cement content leads to a higher UCS of the CPB, and fiber can improve the UCS. The average values of the UCS are also presented in Figure 6: the red dots denote the fiber-reinforced CPB and the blue dots indicate the unreinforced CPB. The respective fitting lines show that the UCS increased exponentially with increasing cement content. Two exponential fitting equations of the UCS, as in Equation (4), of the cement content were obtained with the goodness of fit values of *R*^2^ = 0.9562 and *R*^2^ = 0.9600 for the fiber-reinforced and unreinforced CPB. The *R*^2^ is defined in Equation (5), and the closer *R*^2^ is to 1, the better the formula fits [43,44,45]. The results indicate that increasing the cement content is more conducive to the reinforcement of the fiber effect for the strength of the CPB. More cement could generate more hydration gel, and viscous hydration gel could anchor the fibers more effectively. The fiber and cement type play complementary roles in enhancing the strength of the CPB.

(4) $$\left\{\begin{array}{c} Fiber-reinforced:y=0.1584e^{0.0921\;x}\\ Unreinforced:y=0.0775e^{0.0973\;x} \end{array}\right.$$

where *y* is the UCS of the CPB, MPa, and *x* is the cement content, wt.%.

(5) $$R^{2}=1-\frac{\sum_{i=1}^{n}{\left(y_{i}^{*}-y_{i}\right)}^{2}}{\sum_{i=1}^{n}{\left(y_{i}-\bar{y_{i}}\right)}^{2}}$$

where *n* is the number of data sets; $y_{i}^{*}$ and $y_{i}$ are the estimated and measured UCS values of the *i*th data set.

### 3.3. Estimating the UCS_fiber-reinforced_ with the UCS_unreinforced_

**Figure 7:**
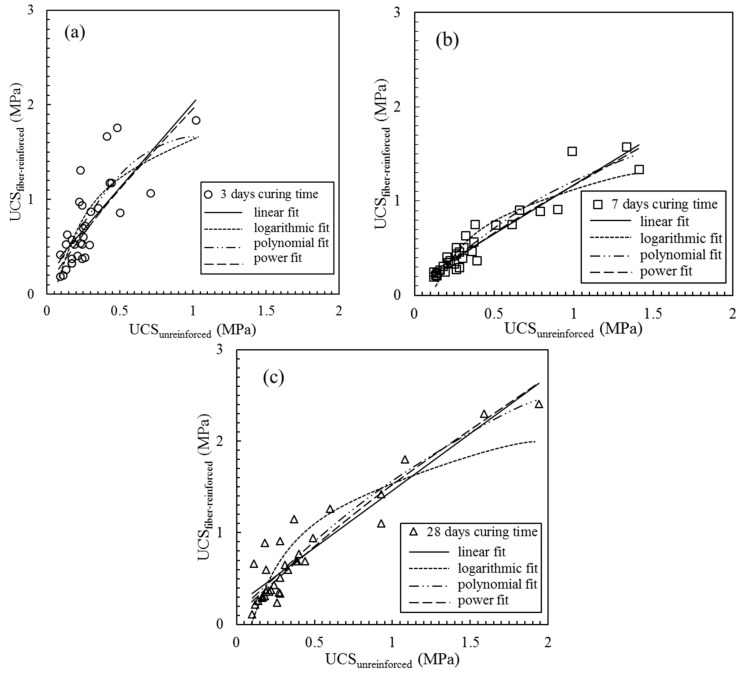
The fitting curves of UCS_fiber-reinforced_ as a function of UCS_unreinforced_ with different curing times: (**a**) 3 days curing time; (**b**) 7 days curing time; (**c**) 28 days curing time.

A total of 96 sets of UCS data of fiber-reinforced and unreinforced CPB are shown in Figure 7, and the UCS of the fiber-reinforced CPB as a function of the UCS of the unreinforced CPB was studied by data fitting. Four different data fitting methods were executed with Excel software, including the linear, logarithmic, polynomial and power fitting methods. Although each fitting curve had a similar trend, there were differences in the fitting curves at each curing age due to differences in the data and fitting accuracy. All the fitting formulas and corresponding goodness of fit values are presented in Table 4, and the best performing formula could be used to estimate the UCS of the fiber-reinforced CPB. As shown in Figure 7 and Table 4, the power fitting method obtained the best goodness of fit value with *R*^2^ = 0.6405 at a curing time of 3 days. At 7 and 28 days of curing, the four fitting formulas all had high fitting accuracies with the goodness of fit values greater than 0.85 and 0.72, respectively. The best fitting accuracy at 7 and 28 days of curing was obtained through the polynomial fitting, with *R*^2^ values of 0.9086 and 0.8766, respectively. Therefore, the three corresponding formulas in Table 4 can be used as a method to estimate the UCS of the fiber-reinforced CPB through the UCS of the unreinforced CPB.

**Figure 8:**
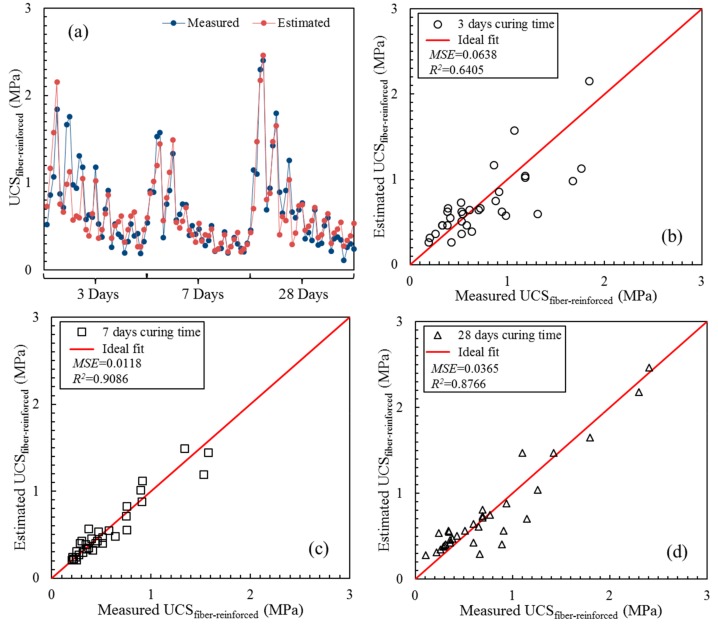
Estimated value of UCS_fiber-reinforced_ and its regression as a function of the measured values with different curing times: (**a**) measured values and estimated values; (**b**) 3 days curing time; (**c**) 7 days curing time; (**d**) 28 days curing time.

Figure 8 illustrates the plots of the estimated values of the fiber-reinforced UCS that were calculated with the best fitting equations shown in Table 4 against the measured values of the fiber-reinforced UCS. Figure 8a shows that three main parts corresponded to UCS values from three different curing ages that exhibited distinctive patterns. The mean squared error (*MSE*) and goodness of fit (*R*^2^) values were implemented as indices of the accuracy of evaluation. Contrary to *R*^2^, the closer *MSE* is to zero, the higher the accuracy [46]. The *MSE* can be calculated by Equation (6). As shown in Figure 8b, the *MSE* and *R*^2^ values of the fit between the estimated value and measured value of the fiber-reinforced CPB at 3 days of curing were 0.0638 and 0.6011, respectively. The discrete distribution of data points near the ideal fit line indicated that the UCS estimation was not very ideal. However, the UCSs of the fiber-reinforced CPB at 7 and 28 days of curing showed good estimation results. Figure 8c,d show that most of the data were distributed in a small error area close to the ideal fit line. The *MSE* and *R*^2^ values of the UCS at 7 days of curing were 0.0118 and 0.9086, respectively. The *MSE* and *R*^2^ of the UCS at a curing time of 28 days were 0.0365 and 0.8766, respectively.

(6) $$MSE=\frac{1}{n}\;\overset{n}{\underset{i=1}{\sum }}{\left(y_{i}^{*}-y_{i}\right)}^{2}$$

The analysis’ results demonstrated that the UCS of the fiber-reinforced CPB after 3 days of curing was accurately estimated and represents a comprehensive influence of hydration reaction intensity, fiber reinforcement and sulfate attack. Thus, it could be used to estimate the material properties.

### 3.4. Fiber Parameters’ Effect on the UCS of CPB with Cement Less Than 13 wt.%

**Figure 9:**
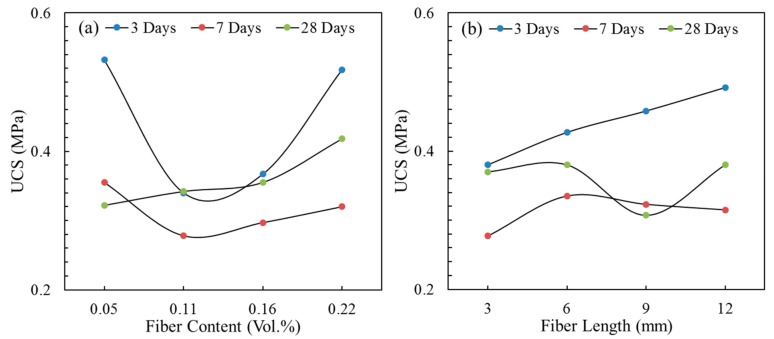
The effects of the fiber parameters on the UCS: (**a**) fiber content; (**b**) fiber length.

Figure 9 shows the UCS of the fiber-reinforced CPB with different fiber contents and fiber lengths. Although fibers can enhance the compactness of the CPB matrix, the new weak structural surface formed by the fibers in the matrix also has a side effect on the strength of the CPB. A higher fiber consumption and longer fiber length do not necessarily result in a higher CPB strength. Therefore, as shown in Figure 9, the CPB strength was different for the fiber parameters with curing ages. At 3 and 7 days of curing, with the increase in the fiber content, the UCS showed a decreasing trend. In contrast, the UCS increased slowly with increasing fiber content at 28 days of curing. Considering both trends, fiber content of 0.22 vol.% was a relatively better choice in this study. For the fiber length, with constant fiber content, the change in the fiber length led to a change in the number of fibers, the contact area and the fiber distribution uniformity. 

In Figure 9b, the UCS increased monotonously with increasing fiber length at a curing age of 3 days; the UCS first increased and then decreased with increasing fiber length at 7 days of curing; at 28 days of curing, the UCS was basically the same except that it was lower for the 9 mm fiber length. There was no clear rule for the influence of the curing age, and the fiber length selection should be based on the strength requirements at different curing ages in the engineering applications.

**Figure 10:**
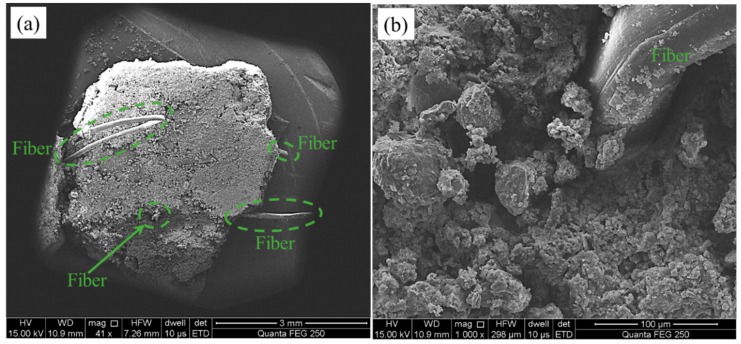
SEM micrographs of the fiber-reinforced CPB with different scales: (**a**) 3 mm; (**b**) 100 µm.

Figure 10 shows the SEM micrographs of the fiber-reinforced CPB. As shown in Figure 10a, several fibers could be distinguished, and their independent distributions showed that the PP fibers had excellent dispensability during the preparation of the CPB. Besides, the fibers exposed on the outside of the CPB and buried in the CPB also illustrated that pull-out and pull-off were the main forms of fiber failure, respectively. During the compression process, the fibers were passively deformed and absorbed energy, thereby enhancing the strength of the CPB. Some of the fibers were not strongly bonded to the CPB matrix, and therefore they deformed and slid under the action of the external force; finally, the fibers were pulled out. Other fibers behaved differently because they were firmly anchored in the CPB matrix; they could not move under the action of the external force and broke when the deformation exceeded the limit. Therefore, the process of fiber interaction with the CPB matrix is the key to the strength of the fiber-reinforced CPB, and this type of interaction can also be explained in Figure 10b. The fibers were embedded in the CPB matrix, and the tailing particles adhered to the surfaces of the fibers under the encapsulation of hydration products such as hydration gel. Under this interaction, the fibers could enhance the compactness of the CPB, and the fibers fixed by bonding could fully exert their reinforcing effect. As a result, the fiber-reinforced CPB had a higher UCS than the unreinforced CPB.

### 3.5. Estimation of the UCS_fiber-reinforced_ Based on Composite Mechanics

From the above analysis, the PP fiber had a significant reinforcement effect on the UCS of the CPB, and the effect was different for different fiber contents and fiber lengths. Therefore, a UCS calculation equation that could quantify the fiber reinforcement needed to be developed. The fiber-reinforced CPB can be considered a composite material composed of multiple phases. It is assumed that the fibers are evenly distributed in the CPB matrix, and the fiber-reinforced CPB is anisotropic material. Its mechanical properties can be considered as the result of the superposition of the mechanical properties of the fibers and unreinforced CPB, and this method has been used to evaluate the UCS of concrete reinforced by steel fibers [35]. Based on the aforementioned test data, a linear relationship between the UCS and fiber reinforcement index (*λ*), which can reflect the comprehensive characteristics of the fiber, is proposed by considering a positive hybrid effect as follows:

(7) $$\sigma_{fiber}=\sigma_{no-fiber}\;\left(1+\alpha \lambda \right)+\beta$$

(8) $$\lambda =V\times \frac{l}{d}$$

where *σ*_fiber_ is the UCS of the fiber-reinforced CPB, MPa; *σ*_no-fiber_ is the UCS of the unreinforced CPB, MPa; *λ* is the PP fiber reinforcement index as shown in Table 5; *V* is the volume fraction of the PP fiber, vol.%; *l*/*d* is the aspect ratio of the PP fiber; *l* is the length of the PP fiber, *d* is the diameter of the PP fiber, mm; *α* is the influence factor of the fiber; *β* is a constant.

**Figure 11:**
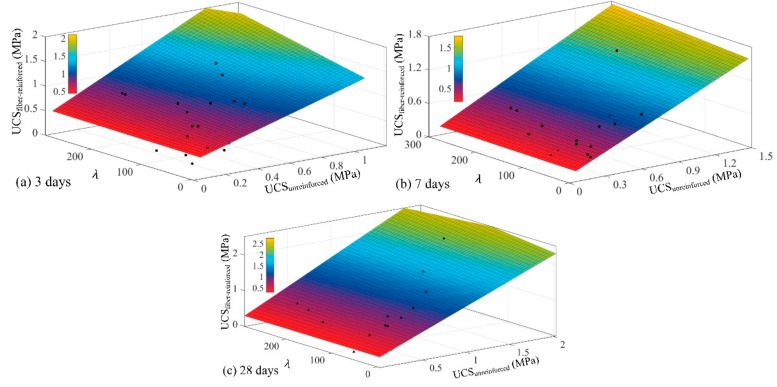
The fitting formula of UCS_fiber-reinforced_ based on composite mechanics with different curing times: (**a**) 3 days curing time; (**b**) 7 days curing time; (**c**) 28 days curing time.

Then, MATLAB was used to establish the fiber-reinforced UCS estimation equation, and three surface equations related to the UCS and fiber reinforcement index were obtained, as shown in Figure 11 and Table 6. Additionally, the fitting curves had a high fitting accuracy at 7 and 28 day curing ages, with the goodness of fit values of 0.9016 and 0.8497, respectively. The equation surface at 3 days of curing exhibited a steep development trend under the action of fibers and *R*^2^ = 0.5304. In addition, as with the quantitative representation of the fiber reinforcement index (*λ*), the greater the value of constant *α*, the greater the contribution of the fibers to the UCS of the CPB. As shown in Table 6, the values of *α* were 0.2072, 0.0408 and 0.0725 at 3, 7 and 28 days of curing, respectively. The highest *α* value at a curing time of 3 days indicates that fibers played a more fully reinforcing role at this curing age. This result is also consistent with the conclusion that the UCS enhancement ratio is higher at 3 days of curing, as summarized in Figure 3.

**Figure 12:**
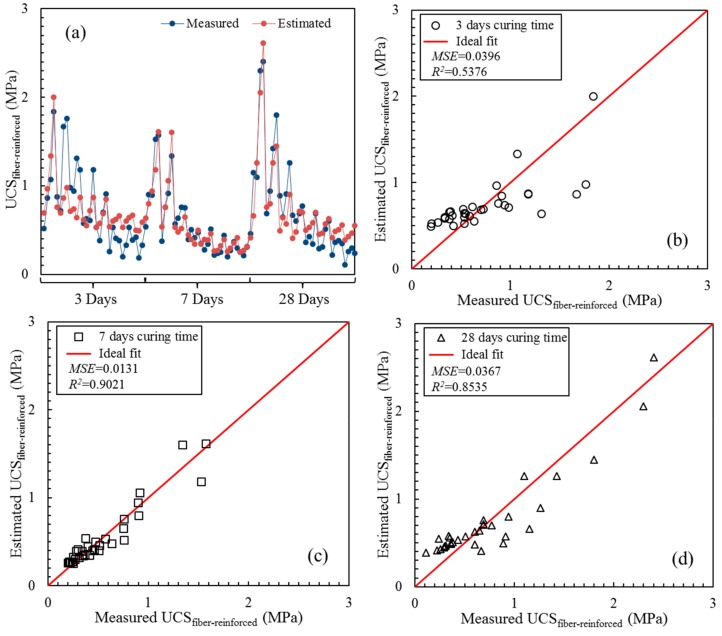
Estimated value of the UCS_fiber-reinforced_ based on composite mechanics and its regression with measured values with different curing times: (**a**) measured values and estimated values; (**b**) 3 days curing time; (**c**) 7 days curing time; (**d**) 28 days curing time.

Figure 12 presents the plots of the estimated values of the fiber-reinforced UCS calculated with the fitting formula in Table 6 against the measured values of the fiber-reinforced UCS based on composite mechanics. The difference between the estimated and measured values of the fiber-reinforced UCS shown in Figure 12a is more significant at 3 days of curing. The *MSE* values at 3, 7 and 28 days of curing were 0.0396, 0.0131 and 0.0367, respectively. The *R*^2^ values at 3, 7 and 28 days of curing were 0.5304, 0.9016 and 0.8497, respectively. Both the *MSE* and *R*^2^ indicated that the fitting formula in Table 6 could be accurate for estimating the UCS of the fiber-reinforced CPB. Figure 12c,d also illustrate that data were concentrated in the vicinity of the ideal fit line. Although the evaluation accuracy was slightly poor, the fitting formula was still instructive for the UCS design of fiber-reinforced CPB at 3 days of curing. Studies have shown that the UCS estimation equation of fiber-reinforced CPB based on composite mechanics can reliably estimate the UCS, and can more clearly quantify the reinforcement effect of the fiber.

### 3.6. Economic and Application Analysis

**Figure 13:**
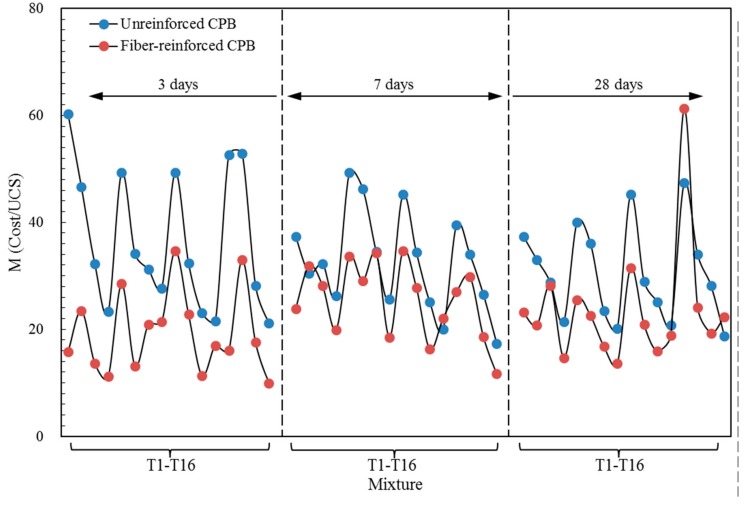
The ratio of materials cost to UCS of CPB.

Comparative analysis of the changes of UCS and materials cost after adding PP fiber with different proportions in Table 7. The costs of materials for unreinforced and fiber-reinforced CPB are 4.74–8.15 $/m^3^ and 5.37–10.15 $/m^3^, respectively. For general mines in China, the cost for producing backfill is a large expenditure, and there will be an increase in cost with fiber-adding when the proportion of CPB is not changed. For example, take the data of mixture T2 shown in Table 7: with the addition of PP fiber, the materials cost increases from 7.93 $/m^3^ to 8.93 $/m^3^, with an increased ratio of 12.61%. If the aim is to keep the UCS as unreinforced CPB, the addition of fiber can reduce the consumption of cement, and the cost to prepare CPB with fibers is cheaper than that without fibers. Take the UCS of CPB mixture T14 with reducing cement consumption 6 wt.% and increasing fiber content 0.16 vol.%. It is basically the same as that of mixture T2 (in Figure 3), and the materials cost is reduced by 1.67 $/m^3^. Those results illustrated that fibers could improve the strength of the CPB and provide potentially significant cost savings. The ratio of materials cost to UCS (M = Cost/UCS) of CPB was calculated and represented in Figure 13, and the results show that adding fiber can effectively reduce the materials cost of CPB to form unit strength with the average decrease ratio of 32.35%. The optimum mixture of the ratio of materials cost to UCS is mixture T4 with a value of 9.94. The addition of fiber increases the materials cost when the CPB proportion is the same; however, its compressive reinforcement effect is more significant.

**Figure 14:**
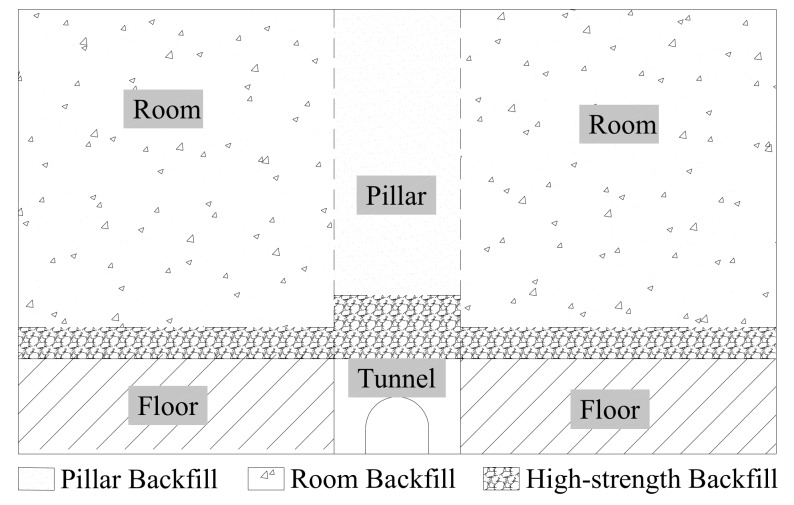
Backfill stopes structure.

It is reasonable and effective to apply low strength, fiber-reinforced CPB as a kind of controlled low-strength material (CLSM). CLSM is a cement-based material mainly used for backfilling with a UCS below 8.3 MPa, and the UCS usually varies depending on project requirements [47,48,49], being approximately 0.7–8.3 MPa for structural fills, approximately 2.8–8.3 MPa for pavement bases and approximately 1.38–2.07 MPa for tunnel shafts and sewer fills. There are no strict regulations for mines to stop backfills based on different functions and applications, and an adequate UCS is approximately 0.7–2.0 MPa [40]. Deng et al. [12] reported that a UCS of approximately 0.08–0.52 MPa can meet the requirements for different mining purposes. In mining, some previous studies have suggested that the strength of the free-standing wall of exposed backfill faces is often up to 1 MPa during pillar recovery [50]. For mine pillar backfill in engineering, despite the high requirements for the roof and floor, the UCS of the pillar stopes is lower. In a two-step pillar recovery, the CPB for the pillar stopes backfill is only required to meet a self-supporting requirement, as shown in Figure 14. Therefore, based on economic and safety considerations, the low-strength CPB in this study can meet the backfill requirements of the pillar stopes. After adding fiber, the CPB with a UCS close to 1 MPa can be considered for filling the room stopes. Besides this, excavation is also a property of the CLSM; a UCS below 0.7 MPa permits easy excavation with digging equipment [47], and the CPB studied in this project meets the excavation requirements. Although mine stopes usually do not need to be excavated after being filled, CPB, as a cement material, will also have some use in construction, pavement bases, tunnels and other fields.

This paper mainly studied the utilization of lead-zinc tailings as raw materials of CPB, focusing on the effect of adding PP fiber on the properties of CPB. Many research results have been achieved in this study, but there are still some limitations to the CPB application. First of all, there are many kinds of fibers, such as steel, glass and carbon fibers, which have been used in the field of concrete [51,52]. It is necessary to study the influence of multi-fiber types and fiber parameters on the CPB. Besides, the effect of tailings and binder materials on CPB is also diverse. The composition, particle size and dosage of a tailing, and the type, dosage and grade of a binder have significant effects on the properties of CPB [53,54,55]. The mechanical effect of more kinds of fibers on CPB made of different types of raw materials should be further studied.

## 4. Conclusions

In this study, a series of experimental investigations were conducted to investigate the reinforcement effect of fibers on UCS of CPB, and nonlinear UCS fitting formulas were proposed to estimate the UCS of the fiber-reinforced CPB. The conclusions can be summarized as follows:

(1) The CPB prepared with a lower amount of cement in this study had a lower UCS, and the UCS of the unreinforced CPB was lower than 0.4 MPa. The UCS of CPB was improved significantly but did not exceed 1.0 MPa. Besides, increasing the cement content could effectively improve the strength of the CPB, and the fiber and cement played complementary roles in enhancing the strength of the CPB.
(2) In this study, the expansion of CPB caused by sulfate attack due to the fibers resulted in the material not being able to fully exert its pressure resistance effect after 3 days of curing. The macroscopic failure modes indicated that the fibers could prevent the formation of large tensile cracks and shear cracks. The SEM tests further showed that the fibers mainly enhanced the UCS of the CPB with pull-out and pull-off failure modes.
(3) The linear and polynomial fitting function could characterize the relationship between the UCS of the fiber-reinforced and unreinforced CPB at the three different curing ages. The nonlinear UCS estimation equation of the fiber-reinforced CPB based on composite mechanics could reliably be used to estimate the UCS and more clearly quantify the reinforcement effect of the fibers with fiber reinforcement index *λ*.
(4) Both estimation results indicated that the UCS of CPB could be estimated accurately. Furthermore, the high fiber reinforcement index *λ* value indicated that fibers played a more fully reinforcing role at 3 day curing age.

Based on the above considerations, the fiber-reinforced CPB can be more widely used in mine backfills. The latter is also an important direction for future research of wide-ranging material applications.

## Figures and Tables

**Table 1:** Main properties of tailings and polypropylene fiber.

Material	Property	Value	Unit
Tailings	Bulk Density (*ρ*)	1.28	t/m^3^
	Particle Density (*ρ_s_*)	3.34	t/m^3^
	Osmotic Coefficient (*K*)	3.10e-06	cm/s
	*D*_50_	25.31	µm
	Uniformity Coefficient (*C_u_* ^1^)	10.30	-
	Curvature Coefficient (*C_c_* ^2^)	0.91	-
	SiO_2_	27.10	%
	Al_2_O_3_	9.11	%
	Fe_2_O_3_	23.70	%
	MgO	1.40	%
	CaO	22.60	%
	K_2_O	2.19	%
	S^2−^	11.90	%
Polypropylene fiber	Density	910	kg/m^3^
	Shape	Fascicular monofilament	-
	Dispersion	Good	-

^1^$C_{u}=D_{60}/D_{10}$; ^2^$C_{C}={\left(D_{30}\right)}^{2}/\left(D_{10}\times D_{60}\right)$.

**Table 2:** Orthogonal table of *L*_16_ (4^4^) for experiment design.

Mixture	Cement Content (A) ^1^ (wt.%)	Solid Mass Concentration (B) ^2^ (wt.%)	Fiber Content (C) ^3^ (vol.%)	Fiber Length (D) (mm)
T1	13	60	0.05	3
T2	13	62	0.11	6
T3	13	64	0.16	9
T4	13	66	0.22	12
T5	10	60	0.11	9
T6	10	62	0.05	12
T7	10	64	0.22	3
T8	10	66	0.16	6
T9	8	60	0.16	12
T10	8	62	0.22	9
T11	8	64	0.05	6
T12	8	66	0.11	3
T13	7	60	0.22	6
T14	7	62	0.16	3
T15	7	64	0.11	12
T16	7	66	0.05	9

^1^ Relatively to the tailing weight; ^2^ Solid mass includes tailing + cement + fiber; ^3^ Volume ratio of fibers to the slurry.

**Table 3:** Orthogonal analysis of ranges of the UCS of CPB (MPa).

Curing Age	Level	Cement Content (A) ^1^ (wt.%)	Solid Mass Concentration (B) ^2^ (wt.%)	Fiber Content (C) ^3^ (vol.%)	Fiber Length (D) (mm)
3 Days	*k*_1_	0.630	0.352	0.532	0.380
	*k*_2_	0.395	0.357	0.340	0.427
	*k*_3_	0.363	0.492	0.367	0.458
	*k*_4_	0.370	0.555	0.518	0.492
	*R_j_*	0.267	0.203	0.192	0.112
	Significance	A>B>C>D			
7 Days	*k*_1_	0.370	0.255	0.355	0.277
	*k*_2_	0.287	0.250	0.278	0.335
	*k*_3_	0.285	0.318	0.297	0.323
	*k*_4_	0.307	0.427	0.320	0.315
	*R_j_*	0.085	0.177	0.077	0.058
	Significance	B>A>C>D			
28 Days	*k*_1_	0.455	0.245	0.322	0.370
	*k*_2_	0.427	0.340	0.342	0.380
	*k*_3_	0.328	0.383	0.355	0.307
	*k*_4_	0.227	0.470	0.418	0.380
	*R_j_*	0.228	0.225	0.096	0.073
	Significance	A>B>C>D			

^1^ Relatively to the tailing weight; ^2^ Solid mass includes tailing + cement + fiber; ^3^ Volume ratio of fibers to the slurry.

**Table 4:** Fitting methods, fitting formulas and *R*^2^ values.

Curing Age	Fitting Method	Fitting Formula	*R* ^2^
3 days	Linear	*σ*_fiber_ = 1.7779 × *σ*_no-fiber_ + 0.2357	0.5849
	Logarithmic	*σ*_fiber_ = 0.6271 × ln(*σ*_no-fiber_) + 1.6316	0.6200
	Polynomial	*σ*_fiber_ = −1.6529 × (*σ*_no-fiber_)^2^ + 3.3925 × *σ*_no-fiber_ − 0.0317	0.6331
	Power	*σ*_fiber_ = 2.1171 × (*σ*_no-fiber_)^0.8580^	0.6405
7 days	Linear	*σ*_fiber_ = 1.0406 × *σ*_no-fiber_ + 0.1321	0.8969
	Logarithmic	*σ*_fiber_ = 0.4979 × ln(*σ*_no-fiber_) + 1.1287	0.8523
	Polynomial	*σ*_fiber_ = −0.3319 × (*σ*_no-fiber_)^2^ + 1.5000 × σ_no-fiber_ + 0.0365	0.9086
	Power	*σ*_fiber_ = 1.1785 × (*σ*_no-fiber_)^0.8067^	0.9009
28 days	Linear	*σ*_fiber_ = 1.2484 × *σ*_no-fiber_ + 0.2131	0.8676
	Logarithmic	*σ*_fiber_ = 0.6835 × ln(*σ*_no-fiber_) + 1.5405	0.7906
	Polynomial	*σ*_fiber_ = −0.2487 × (*σ*_no-fiber_)^2^ + 1.6945 × *σ*_no-fiber_ + 0.1110	0.8766
	Power	*σ*_fiber_ = 1.5213 × (*σ*_no-fiber_)^0.8262^	0.7297

**Table 5:** Polypropylene (PP) fiber reinforcement index (*λ*).

CPB Sets	*V*^1^ (%)	*l*/*d*^2^	*λ* ^3^	CPB Sets	*V*^1^ (%)	*l*/*d*^2^	*λ* ^3^
1	0.11	157.89	0.17	17	0.05	157.89	0.09
2	0.22	315.79	0.69	18	0.11	315.79	0.35
3	0.33	473.68	1.56	19	0.16	473.68	0.78
4	0.44	631.58	2.78	20	0.22	631.58	1.39
5	0.22	473.68	1.04	21	0.11	473.68	0.52
6	0.11	631.58	0.69	22	0.05	631.58	0.35
7	0.44	157.89	0.69	23	0.22	157.89	0.35
8	0.33	315.79	1.04	24	0.16	315.79	0.52
9	0.33	631.58	2.08	25	0.16	631.58	1.04
10	0.44	473.68	2.08	26	0.22	473.68	1.04
11	0.11	315.79	0.35	27	0.05	315.79	0.17
12	0.22	157.89	0.35	28	0.11	157.89	0.17
13	0.44	315.79	1.39	29	0.22	315.79	0.69
14	0.33	157.89	0.52	30	0.16	157.89	0.26
15	0.22	631.58	1.39	31	0.11	631.58	0.69
16	0.11	473.68	0.52	32	0.05	473.68	0.26

^1^ Volume fraction of the PP fiber; ^2^ aspect ratio of the PP fiber; ^3^ PP fiber reinforcement index.

**Table 6:** The results of the estimated UCS_fiber-reinforced_ based on composite mechanics.

Curing Ages	Fitting Formula	*R* ^2^	*α*	*β* (×10^−2^)
3 d	*σ*_fiber_ = *σ*_no-fiber_ (1 + 0.2072 × *λ*) + 0.3097	0.5304	0.2072	0.3097
7 d	*σ*_fiber_ = *σ*_no-fiber_ (1 + 0.0408 × *λ*) + 0.1316	0.9016	0.0408	0.1316
28 d	*σ*_fiber_ = *σ*_no-fiber_ (1 + 0.0725 × *λ*) + 0.2849	0.8497	0.0725	0.2849

**Table 7:** The influence of adding fiber on the materials cost of CPB.

Mixture	Cement Content (wt.%)	Fiber Content (vol.%)	Materials Cost ($/m^3^)	
			No-Fiber	Fiber
T1	13	0.05	7.85	8.35
T2	13	0.11	7.93	8.93
T3	13	0.16	8.06	9.57
T4	13	0.22	8.15	10.15
T5	10	0.11	6.41	7.41
T6	10	0.05	6.49	6.99
T7	10	0.22	6.57	8.57
T8	10	0.16	6.65	8.15
T9	8	0.16	5.43	6.93
T10	8	0.22	5.51	7.51
T11	8	0.05	5.53	6.03
T12	8	0.11	5.61	6.62
T13	7	0.22	4.74	6.74
T14	7	0.16	4.76	6.26
T15	7	0.11	4.78	5.79
T16	7	0.05	4.87	5.37
